# Caution required when relying on a colleague's advice; a comparison between professional advice and evidence from the literature

**DOI:** 10.1186/1472-6963-5-59

**Published:** 2005-08-31

**Authors:** Frederieke Schaafsma, Jos Verbeek, Carel Hulshof, Frank van Dijk

**Affiliations:** 1 Coronel Institute for Occupational and Environmental Health, Academic Medical Centre, PO Box 22700, 1100 DE Amsterdam, The Netherlands; 2 Cochrane Collaboration Occupational Health Field, Finnish Institute of Occupational Health, PO Box 93, 70701 Kuopio, Finland

## Abstract

**Background:**

Occupational Physicians rely especially on advice from colleagues when answering their information demands. On the other hand, Evidence-based Medicine (EBM) promotes the use of up-to-date research literature instead of experts. To find out if there was a difference between expert-based practice and EBM we compared professional advice on occupational health topics with best evidence from the literature.

**Methods:**

We asked 14 occupational physicians to consult their usual information sources on 12 pre-conceived occupational health problems. The problems were presented in the form of case vignettes which contained sufficient clinical information to be used by the occupational physicians for the consultation of their experts. We had searched the literature for the best available evidence on the 12 problems, which made it possible to answer the clinical questions with a clear yes or no.

**Results:**

The cases could be used by the occupational physicians as arising from their own practice. All together the occupational physicians consulted 75 different experts. Almost half of the consulted experts were near colleagues, 10% were industrial hygienists, 8% medical specialists and the rest had a varied background. Fifty three percent (95% confidence interval 42% to 65%) of all professional advice was not in line with the research literature. In 18 cases (24%) professional advice explicitly referred to up-to-date research literature as their used source. These cases were substantially less incorrect (17%) than advice that had not mentioned the literature as a source (65%) (difference 48%, 95% Confidence Interval from 27% to 69%).

**Conclusion:**

Advice that occupational physicians routinely get in their daily practice differs substantially from best evidence from the literature. Occupational physicians who ask professional advice should always ask about the evidence of this advice.

## Background

Occupational physicians (OPs) in their daily routine are confronted with a large variety of occupational health problems. From previous research we know that in attending these problems OPs mostly rely on their own experience and on information from consulting an experienced colleague [1]. On the contrary, Evidence-Based Medicine proposes to use evidence from the up-to-date research literature as most reliable source. Reasons for OPs to still prefer working experience- or authority-based are the relatively easy way to obtain and the attributed validity of the information. Evidence-based medicine, although much-supported, is still not a customary way for occupational physicians (OPs) to address problems that arise in their daily work [2]. OPs like other physicians do not quite see its benefits.

Relying on your own or on others' expertise knows some drawbacks. For example, Slawson et al described that the information can be out of date and that there could be the matter of reverse gullibility [3-5]. In this study we want to challenge the belief of OPs that asking for professional advice from a colleague, even if this colleague is considered an expert on the subject, is a good source for information. We will compare professional advice given by experts to answers based on best evidence derived from the literature.

## Methods

We asked a convenience sample of fourteen acquainted OPs working scattered over four different regions of the Netherlands to collect data for us. Our main criteria to ask a physician to participate were that he or she had to be professionally sufficiently experienced. Next, we took care that there was variation in location to avoid the situation that the same professional expert would be asked about the same case vignette by different OPs. Even though we tried to vary age, gender and professional experience, the majority was over 40, male and had a long standing professional experience and three OPs had achieved a doctor's degree. (Table 1) All OPs were considered experienced and professionally motivated, and agreed to participate. The OPs were requested to obtain two professional advices on each of three case vignettes which would lead to a maximum of 84 cases. To be able to show that a relevant 15% of the answers would not be in line with the literature with α = 0.05 and β = 80% we would need about 53 cases. A professional advice was defined as an advice from a person who was considered by the OP to be an expert on the subject and who would also be consulted in the normal course of daily routine.

**Table 1:** Personal characteristics of occupational physicians (N = 14) involved in the study

	N (%)
Age (> 40 years)	10 (71)
Gender (male)	12 (86)
Geographical location	
North	4 (29)
South	3 (21)
West	4 (29)
East	3 (21)
Certified occupational physician	12 (86)
Professional Experience (> 10 years)	12 (86)
Occupational Health Service	
Arbounie	7 (50)
Other	7 (50)
Academic Status (PhD)	3 (21)

Twelve cases were selected on the basis of a clear occupational health problem, resemblance to daily practice for an OP and assumption that there would be sufficient literature (Table 2, See Additional file 1). The cases represent a broad variety of occupational health practice ranging from return to work interventions in workers with musculoskeletal disorders to the causality of stress in case of a myocardial infarction. The case vignettes ended in a clear clinical question that could be answered by a simple yes or no. For example, 'does continuous years of work stress increase the risk of a myocardial infarction?' and 'is it useful to take melatonin to prevent jetlag?'

**Table 2:** Summary of the case vignettes and correct evidence-based answer

1.	For a 36-year old caretaker at a secondary school with a lateral ankle ligament rupture treated with tape for three weeks, is it safe to resume work? Yes
2.	Can a rash on the inside of the forearm of a 43-year old production worker be caused by exposure to PVC during the production of bathroom doors? Yes
3.	Can continuous years of work stress be the cause of a cardiac infarct in a 54-year-old bank employee with only a slightly raised cholesterol level? Yes
4.	For a 38-year old laboratory worker with epicondylitis lateralis, does electro shock wave therapy (ESWT) produce better results in reducing complaints than conventional treatment with physiotherapy and analgesics? No
5.	Is a 38-year old sewage worker subject to a higher risk of contracting Hepatitis A as a result of occupational exposure? No
6.	For a 48-year old archivist with extrinsic allergic alveolitis, is it useful to investigate the archive more closely for fungal cultures as a possible cause for the lung disease? Yes
7.	Is it safe for a 42-year old parking attendant suffering from a whiplash as a result of a car accident to return partially to work after some 10 days? Yes
8.	Is Cognitive Behaviour Treatment more effective than other therapies for a 45-year old teacher diagnosed with burnout? Yes
9.	Is it effectively useful to take melatonin to prevent jetlag for workers of an ICT firm travelling to Asia? Yes
10.	For a 45-year old female teacher diagnosed with mild depression, is St. John's Wort more effective than placebo? Yes
11.	Does a return to his physically demanding work after an operation on a lumbal hernia nuclei pulposi in a 45-year-old carpenter, six weeks after the operation, give a higher risk of a recurrence than returning to only light work? No
12.	Can a 42-year old male nurse, working on the ambulances safely return to full time work three weeks after his inguinal hernia operation? Yes

The OPs were asked to draw their own conclusion on the case vignettes and to provide the professional advice of all the experts that were consulted. The OP could decide for himself whether or not to rely on the advice received. All cases had to be advised on by the experts with yes or no accompanied by a motivation for the answer. The experts were kept unaware by the consulting OP that the cases presented were fictive.

These professional advices were compared to evidence from the literature in the form of a critically appraised topic (CAT). Critically appraised topics are considered as the best way to retrieve an answer to a question arising from practice from the literature. We followed the guidelines for making critically appraised topics as formulated by Sacket et al.[6] We used Medline, the Cochrane Library and the Dutch clinical guideline database (CBO) to search for relevant evidence to the clinical questions. We used the best available evidence that we could find on a certain topic. In three cases we could use a Cochrane systematic review, in four cases we could use a systematic review and in 5 cases we relied on original studies as the best evidence because no systematic review was available. We felt that for none of the cases the evidence was novel or surprising, but that the available recent literature all pointed in the same direction. All CATs are described in the appendix together with the search strategy and the evidence that was used to answer the clinical question. [See Additional file 1]

A professional advice was considered correct if both the 'yes or no answer' and the motivation were in line with the evidence from the literature as summarised in the CAT. The conclusions of the OPs were assessed only by their 'yes or no answer'.

The first two authors (FS and JV) checked and evaluated both the professional advices and the answers from the OPs separately. We measured the proportion of advices and answers that were not correct.

## Results

The occupational physicians consulted 84 different experts of which 75 answered (89% response; 75 out of 84). This resulted in 39 answers to the case vignettes from the 14 participating OPs (93% response; 39 out of 42) on the 12 cases. All cases were perceived as being from daily practice by both the OPs and the consulted experts. Each individual case was advised on at least five times by an expert, except for one case where we had only two advices from experts. Table 3 shows the profession of the consulted experts which are comparable to the type of experts occupational physicians usually consult in daily practice [1]. Most experts were consulted via e-mail (37.3%), by telephone (28.0%) or directly (13.3%). Of the 75 professional advices, 28 (37 %, 95% Confidence Interval from 26% to 48%) were incorrect. If we also took the motivation related to the answers in consideration, 40 answers were incorrect (53%, 95% Confidence Interval from 42% to 65%). Of the 39 conclusions of the OPs, based on the experts' advice 17 (44%, 95% Confidence Interval from 28% to 59%) were incorrect. There was no difference in the rate of incorrect advice per type of profession per consulted expert or per case vignette.

**Table 3:** Frequency of consulted colleagues

Profession of the consulted colleague	Number of consultations N (%)
Occupational Physician	34 (45.3)
Occupational Hygienist	8 (10.7)
Medical Specialist from a local hospital	6 (8.0)
Physiotherapist	6 (8.0)
Professional at a specialized occupational health centre or clinic	6 (8.0)
Psychologist	4 (5.3)
Other	11 (14.7)
Total	75 (100.0)

The motivations of the experts for their advices were based 18 times (24%) on the literature. The rate of incorrect advices by experts was 17% if their advices were explicitly based on the up-to-date research literature versus 65% incorrect if these advices were not based on the literature (difference 48%, 95% Confidence Interval from 27% to 69%).

## Discussion

This is a first empirical study about the difference between research literature and the knowledge of professionals within occupational health. The results substantiate the claim by previous authors that physicians should be more aware of the limited value of the information obtained from experts [3]. Less than half of the given professional advice by experts to a practical occupational health problem was in line with evidence from the research literature.

The strength of our study is that we used the information retrieval process such as it occurs in real daily practice of occupational physicians. From our previous study, we know that the information sources that occupational physicians used in this study do conform to the sources they use in general. About half of them ask a colleague, 20% ask other professionals in the occupational health area and another 20% consults medical specialists or other clinical experts. None of the participants in the study commented on the nature of the cases or the questions asked. They were all perceived as relevant and important for clinical occupational health practice. The occupational physicians were situated in different parts of the country and had similar training as occupational physicians in general. There was an overrepresentation of physicians with a doctor's degree in our sample. This might have positively influenced the results in a way that more academic professionals could have been consulted. In turn, we assume this would have resulted in answers more in line with the literature. However, we did not find indications for such a mechanism. The power of the study was sufficient to show at least a 15% deviance from evidence from the literature. Therefore, we feel that there is no reason to believe that the practice of professional advice studied here is different for other OPs or even in other medical disciplines as argued by various authors. [3-5]

Answers to clinical questions arising from practice should not only depend on the available evidence but also on the clinical situation, the patient's preferences and the resources available. The selected case vignettes all required dichotomous answers from the experts and OPs. This obviously distorts to some extent the clinical reality. However, the decision making was rather obvious in all cases with a clear patient preference, and the cases were perceived as being from daily practice even by the experts who were unaware of the fictive nature. As to the resources available, we considered leaving this open for the consulted expert to resemble daily practice most.

The evidence we used to answer to the cases is a selection following the guidance given by the experts.[6] For most cases we found good systematic reviews which can be considered as high quality evidence. However, in some we had to rely on single original studies that were not always evaluation studies. This leaves some room for discussion about the credibility of the evidence. However, none of the results of the studies used as evidence were really novel or surprising but all were in line with general trends in the literature such as the approach to musculoskeletal diseases or advice about return to work. Moreover, the results were not related to the type of case and therefore not to the quality of the evidence provided.

## Conclusion

Our findings urge for more and better research into professional knowledge management. For now we conclude that better use of the available research literature is possible and should be stimulated among occupational physicians. If professionals considered an expert on the subject, are asked for advice, occupational physicians should still make sure that the expert also provides the evidence for his advice.

## Competing interests

The author(s) declare that they have no competing interests. The views expressed in this article represent those of the authors and are not necessarily the views of the official policy of The Cochrane Collaboration.

## Authors' contributions

JV designed the project plan. FS and JV carried out the project. FS wrote the first draft of the manuscript. All authors commented equally on the project plan and all drafts of the manuscript and read and approved the final manuscript.

## Pre-publication history

The pre-publication history for this paper can be accessed here:



## Supplementary Material

Additional File 1 We give the 12 Critically Appraised Topics that we used as the literature standard to compare the experts' advice with as well as the search strategy and literature references on which the CATs conclusions are based. Click here for file

## References

[B1] Schaafsma FG, Hulshof CT, van Dijk FJ, Verbeek JH (2004). Information demands of Occupational Physicians and their attitude towards Evidence-Based Medicine. Scand J Work Environ Health.

[B2] Verbeek JH, van Dijk FJ, Malmivaara A, Hulshof CT, Rasanen K, Kankaanpaa EE, Mukala K (2002). Evidence-based medicine for occupational health. Scand J Work Environ Health.

[B3] Slawson DC, Shaughnessy AF (1997). Obtaining useful information from expert-based sources. BMJ.

[B4] Antman EM, Lau J, Kupelnick B, Mosteller F, Chalmers TC (1992). A comparison of results of meta-analyses of randomized control trials and recommendations of clinical experts. Treatments for myocardial infarction. JAMA.

[B5] Riffenburgh RH (1996). Reverse Gullibility and Scientific Evidence. Arch Otolarygol Head Neck Surg.

[B6] Sackett DL, Straus SE, Richardson WS, Rosenberg W, Haynes RB (2000). Evidence-based medicine. How to practice and teach EBM.

