# Supplementary material for: Caution required when relying on a colleague's advice; a comparison between professional advice and evidence from the literature
Source: BMC Health Serv Res. 2005 Aug 31;5:59. doi: 10.1186/1472-6963-5-59 (PMC1208884; doi:10.1186/1472-6963-5-59)
Supplement: Additional File 1 — We give the 12 Critically Appraised Topics that we used as the literature standard to compare the experts' advice with as well as the search strategy and literature references on which the CATs conclusions are based. [file 1472-6963-5-59-S1.doc]

CAT I

1. Question:

Is it necessary for someone with a lateral ankle ligament rupture, treated with tape, to avoid putting pressure on the ankle for at least six weeks? Profession: caretaker at a secondary school.

1. Answer: No.

It takes on average six weeks for complete recovery and after two weeks, on average, it is possible to start walking again and return to work.

1. Background:

A 36-year old man is working full time as caretaker at a secondary school. While playing soccer the previous week, he ruptured his lateral ankle ligament. The First Aid personnel treated the injury with tape. This tape must stay in place for six weeks. The patient indicates to the occupational physician that he certainly will not be able to return to work within six weeks. His tasks involve walking around the school building at irregular intervals, and sitting in his office. He is more or less free to choose when he performs these tasks

1. PICO:

P: lateral ankle ligament rupture, I: tape, C: - , O: recovery/return to work

1. Search terms and results:

PUBMED search: "Lateral Ligament, Ankle"[MESH] AND rupture AND tape

Results: 4 articles: 1 systematic review

CBO guidelines: Consensus-based diagnostic strategy and treatment of the acute ankle injury 1998

1. Research and quality:

CBO guidelines: Two RCTs compared different methods of functional treatment with each other. In the first study, three weeks with a plaster cast was compared with six weeks with an air cast and six weeks with tape. In the second study, six weeks with an air cast, six weeks with tape, six weeks wearing a special shoe, and six weeks wearing an elastic sock were compared with each other.

Systematic Review: 9 RCTs are compared with 892 patients

1. Results

CBO guidelines: The average period before return to work for functional treatment with tape is 15 days as opposed to 38 days with plaster. The results in the first RCT of the three groups were similar. The results in the second RCT of the aircast, tape, and shoe were also similar. The elastic sock gave a higher 'giving way' and pain percentage, while the return to work lasted the longest (25 days compared with 16 days in the tape group).

In the summary of the guidelines: with functional treatment with tape, it is possible to walk normally after two weeks.

Systematic review: Comparison of symptoms (amongst other things, swelling) between lace-up ankle support with semi-rigid ankle support: RR 4.2 (95% CI 1.3-14)

Elastic bandage: RR 5.5 (95% CI 1.7-18)

Tape: RR 4.1 (95% CI 1.2-14)

Conclusion with respect to return to work: the use of tape results in a more rapid return to work and sports activities than the use of elastic bandage, but causes more complications (such as skin irritation).

8. References:

1. CBO guidelines: Consensus-based diagnostic strategy and treatment of the acute ankle injury 1998; *The Dutch Institute for Healthcare Improvement*. http://www.cbo.nl/product/richtlijnen/pdf/enkel

2. Kerkhoffs GM, Struijs PA, Marti RK, Blankevoort L, Assendelft WJ, van Dijk CN. Functional treatments for acute ruptures of the lateral ankle ligament: a systematic review.

*Acta Orthop Scand* 2003 Feb; 74(1):69-77

Signed:

Frederieke Schaafsma, Email: [f.g.schaafsma@amc.uva.nl](mailto:f.g.schaafsma@amc.uva.nl)

May 2003

CAT II

1. Question:

Is it possible that a rash on the inside of the forearm of a 43-year old production worker is caused by exposure to PVC in the production of bathroom doors?

2. Answer:

Yes, in the plastics industry, the risk of self-reported rash with exposure to PVC is four times higher than without exposure to PVC. This can also be caused by additives to PVC.

3. Background:

A 43-year old production worker in a manufacturing plant of plastic doors has a rash on the medial side of both forearms. The rash consists of erythema with small vesicles. During the surgery hours of the occupational physician, he asks whether this could be caused by working with PVC. The PVC is delivered in granula form and is moulded into a door with the aid of a cast. He has frequent direct skin contact with the granula. His manager assured him that there was no reason for him to become anxious about working with plastic.

4. PICO

P: 43-year old production worker in a doors/plastic factory. I/E: exposure to PVC. C: no exposure. O: dermatitis / erythema

5. Search terms and results

PubMed: pvc [mesh] AND dermatitis [mesh]

Results: 29 articles, case reports and surveys of companies

6. Research and quality

a. 122 employees from four different companies in the USA were asked to complete questionnaires to determine the risk of skin disorders, and employees were assigned into groups according to exposure, such as exposure to chemicals, or the use of soaps and creams. An Odds Ratio approaching 1 would indicate the greatest chance of bias in the results.

b. In various case studies, the possibility of an allergy to additives such as pyridine and phtalates is indicated.

7. Results

With respect to exposure to PVC there were 10 complaints from a sample of 25 employees, as opposed to 16 of 97 without exposure. The rough OR was 3.38 (95%CI 1.16-9.84). In a logistic regression comparison in which corrections for gender, age, protective creams, cleaning products, and exposure to formaldehyde were made, the OR was 4.08 (95%CI 1.19-14.06).

8. Reference

Socie EM, Gromen KD, Migliozzi AA, Geidenberger CA. Work-related skin disease in the plastics industry. *Am J Ind Med* 1997 May; 31(5):545-50

1. Signed :

April 2003, Jos Verbeek. Email: [j.h.verbeek@amc.uva.nl](mailto:j.h.verbeek@amc.uva.nl).

CAT III

1. Question: Does continuous years of work stress increase the risk of a myocardial infarction?

2. Answer: Yes, corrected for biomedical variables such as smoking, cholesterol, BMI, and hypertension, the HR was 2,2 for the group that experienced a lot of work-related stress with respect to the group that experienced little stress.

3. Background: A 54-year old bank employee attends surgery hours of the company doctor after six weeks of sick leave as a result of his first infarct. The only risk factor he has is a slightly raised cholesterol level. He has discussed this with the family doctor and together they have come to the conclusion that work stress is the most important cause of the infarct. He works in a department that has been constantly under fire for a period of years and where reorganisations have constantly taken place during the previous ten years. A recent PAGO (periodic health appraisal examination) demonstrated high scores on the work stress scale. He asks the company doctor to confirm that his condition is an occupational disease.

4. PICO:

P: 54-year old administrative employee

I/E: exposure to psychosocial stress, in particular work stress

C: little or no exposure to psychosocial stress

O: cardiac infarct

5. Search terms and results:

cardiovascular diseases [mesh] AND stress, psychological [mesh] AND occupational diseases [mesh]; 254 articles, of which no. 12 is used

6. Research and quality

No 12 is a cohort study in Finland among employees at a metal factory with a 25 year follow-up. Stress was measured using validated questions. After correction for occupation and biomedical variables (smoking, cholesterol and hypertension) an elevated risk for the group that scored high for stress variables remained.

7. Results

The Hazard Ratio for dying from cardiovascular diseases was 2,2 (95%BI 1,2-2,4) for the group that was exposed to high work stress with respect to the group that indicated little work stress.

8. Reference

Kivimaki M, Leino-Arjas P, Luukkonen R, Riihimaki H, Vahtera J, Kirjonen J. Work stress and risk of cardiovascular mortality: prospective cohort study of industrial employees*. BMJ*. 2002 Oct 19; 325(7369):857.

9. Signed:

July 2003, Jos Verbeek, [j.h.verbeek@amc.uva.nl](mailto:j.h.verbeek@amc.uva.nl)

CAT IV

1. Question:

Is electro shock wave therapy (ESWT) more effective in reducing complaints resulting from epicondylitis lateralis than conventional treatment with physiotherapy and analgesics?

2. Answer: No.

There are conflicting outcomes in the various studies. No relevant difference has been established between the two treatments. In view of the costs involved, the conventional treatment is still preferred. There is no reason for the employer to choose ESWT in favour of standard therapy with physiotherapy and analgesics.

3. Background:

A 38-year old female employee of a hospital blood collection section has restrictions in using her right arm as a result of epicondylitis lateralis. The employer is willing to offer her ESWT so that she can return to work as soon as possible. He asks the occupational physician for an advice.

4. PICO:

P = 38-year old female employee of a hospital blood collection section with epicondylitis lateralis

I = ESWT

C = conventional therapy

O = reduction in complaints

5. Search terms and results:

Cochrane Library: Shock wave therapy, lateral elbow pain

Three hits, one systematic review

6. Research and quality:

Two trials of ESWT versus placebo have been included in the review. Both trials consisted of comparable populations of patients with chronic complaints that did not respond to conventional therapy. The frequency of ESWT, the dosage and the technique was similar in both trials. The first trial produced significantly better results for ESWT compared with placebo; the second trial produced no significant improvement.

1. Results

After pooling the results of the two trials, the positive significant results of the first trial disappeared. RR for “treatment failure” of ESWT (defined as Roles-Maudsley score of 4) with respect to placebo was 0.40 (95% CI: 0.08-1.91) at six weeks and 0.44 (95% CI: 0.09-2.17) at one year.

8. Reference:

Buchbinder R, Green S, White M, Barnsley L, Smidt N, Assendelft WJ. Shock Wave Therapy for lateral elbow pain. *Cochrane Database Syst Rev*. 2002;(1):CD003524

9. Signed: F.G. Schaafsma, Email: [f.g.schaafsma@amc.uva.nl](mailto:f.g.schaafsma@amc.uva.nl)

June 2003

CAT V

1. Question: Are sewage workers subject to an increased risk of contracting Hepatitis A as a result of occupational exposure?

2. Answer: Exposure to sewage work does not produce a higher risk of a clinically observable form of Hepatitis A. It is possible that the seroprevalence of anti-HbA is increased.

3. Background: A 30-year old employee of a sewage purification installation company asks the occupational physician if he should be vaccinated against Hepatitis A. The employees have been talking about it amongst themselves. After all, all kinds of bacteria and viruses exist in sewage water. While he is performing his work there are spray and spatters, in which these organisms could be living.

4. PICO:

P: 30-year old healthy employee of a sewage water company

I/E: exposure to sewage water

C: no exposure

O: hepatitis A

5. Search terms and results:

Hepatitis A [Mesh] AND sewage [Mesh]

67 articles, of which number 6 is a systematic review

6. Research and quality

In a systematic review, 17 studies were found of which 1 was historical prospective, 15 were cross-sectional and 1 was descriptive.

7. Results

In 4 of the 5 studies that measured a reliable clinical outcome, no relation was found between exposure and the appearance of clinical symptoms of Hepatitis A. In 14 cross-sectional studies and 20 comparisons that measured the seroprevalence of HbA antibodies, there were only three comparisons with a definitely increased odds ratio. In most studies there was a high suspicion of publication bias and confounding.

8. Reference

Glas C, Hotz P, Steffen R. Hepatitis A in workers exposed to sewage: a systematic review.
*Occup Environ Med*. 2001 Dec; 58(12):762-8.

9. Signed

May 2003, Jos Verbeek; [j.h.verbeek@amc.uva.nl](mailto:j.h.verbeek@amc.uva.nl)

CAT VI

1. Question:

Is it useful to screen an archive for fungal cultures as a possible cause for extrinsic allergic alveolitis?

1. Answer:

Yes, if there are damp areas with fungi, there could be a relation. Closer investigation is useful.

1. Background:

A 48-year old archivist has been on sick leave for some time due to lung complaints. His complaints have persisted for the previous 18 months, and he had spent periods sick at home on different occasions. His last official sick leave episode was a month ago. He has been treated by the lung specialist and, after a thorough examination; he has noted a diagnosis of extrinsic allergic alveolitis. A serum test shows a weak positive reaction to cladosporium. The lung specialist phones the occupational physician to ask whether there are indications for investigating his workspace more closely. The occupational physician knows of the existence of an old RI&E (Risk, Inventory, and Evaluation) report from 1999 that states that the archive where he works is very old-fashioned and that the maintenance of the building is far from optimal. There is no further useful information.

1. PICO:

P: man, I: fungus/cladosporium, C:-, O: extrinsic allergic alveolitis

1. Search terms and results:

PubMed search: Alveolitis, Extrinsic Allergic AND Cladosporium

Result: 8 articles, no meta-analysis or review; 3 case reports

Up to Date: reference to Occup Med 1992 Apr-Jun;7(2):271-86 in which there is a description of possible routes of lung infection and the relation with water.

1. Research and quality:

3 case reports of pneumonia.

1. Results

The three case reports describe cases of persons with symptoms of severe pneumonia whereby later research shows that traces of cladosporium were found in the home. Usually as a result of damp, and therefore fungal cultures.

1. Reference:

1. Jacobs RL, Andrews CP. Hypersensitivity pneumonia-non-specific interstitial pneumonia/fibrosis histopathologic presentation: a study in diagnosis and long-term management. *Ann Allergy Asthma Immunol*. 2003 Feb;90(2):265-70.

2. Jacobs RL, Thorner RE, Holcomb JR, Schwietz LA, Jacobs FO.

Hypersensitivity pneumonitis caused by Cladosporium in an enclosed hot-tub area. *Ann Intern Med*. 1986 Aug;105(2):204-6.

3. Schwarz H, Wettengel R, Kramer B. Extrinsic allergic alveolitis in domestic environments (Domestic allergic alveolitis) caused by mouldy tapestry. *Eur J Med Res* 2000 Mar 27;5(3):125

9. Signed: Frederieke Schaafsma, June 2003 Email: [f.g.schaafsma@amc.uva.nl](mailto:f.g.schaafsma@amc.uva.nl)

CAT VII

1. Question:

Is someone suffering from a whiplash as a result of a car accident allowed to return partially to work as a parking attendant after 10 days, depending on his/her complaints?

1. Answer:

Yes, early mobilisation is an aid towards successful recovery from complaints.

1. Background:

A 42-year old male driver of a car was hit from behind by a car travelling at 40 km/hour, where he himself was able to brake in time. He did not lose consciousness. X-CWK showed no abnormality. After two days, he developed serious neck complaints accompanied by headache. Diagnosis of the general physician: whiplash

General physicians’ provisional advice: rest and Ibuprofen. The person involved started moving about after 2-3 days of his own accord and has already performed some neck exercises. He was suggested these exercises by a friend who is a physiotherapist.

He would now like to get back to work partially. His complaints have eased but are still present. He uses Ibuprofen when necessary. His general physician does not support his wishes.

1. PICO:

P: whiplash; I; early mobilisation C; rest O: recovery

1. Search terms and results:

PubMed: Clinical Queries: Whiplash AND systematic review

Result: 29 systematic reviews

1. Research and quality:

A systematic review on the effectiveness of different exercise methods for the treatment of various types of neck complaints. 16 trials have been included. Four RCTs studied whiplash-associated disorders (WAD). One RCT (Mc. Kinney 1989) focused on the effects of early mobilization exercises (6 PEDscore).

1. Results with respect to early mobilization:

Mc. Kinney (1989): 170 pt with WAD, three different groups:

Recovery was significantly better in patients given advice on early mobilisation to do at home, than in other patients (x2 =5, 43 df =1 p= 0,02). Early mobilization was found more effective in reliving neck pain in acute neck sprains, than physiotherapy or rest.

1. Reference:

Sarig-Bahat. H; Evidence for exercise therapy in mechanical neck disorders

*Manual Therapy* (2003) 8 (1), 10-20

9. Signed: Frederieke Schaafsma, Email: [f.g.schaafsma@amc.uva.nl](mailto:f.g.schaafsma@amc.uva.nl) June 2003

CAT VIII

1. Question: Is CBT (Cognitive Behaviour Treatment) more effective than other forms of psychotherapy in the case of burnout?

2. Answer: Cognitive Behaviour Treatment is more effective than relaxation techniques, multi-modal interventions, and organisation-focused interventions for employees exposed to stress.

3. Background: A 45-year old teacher is complaining about tiredness, sleeping badly, concentration disorders, having the feeling that he isn’t performing any more, and is not liking the students at school. He is diagnosed by the occupational physician as having a burnout. The teacher agrees and enquires about the best therapy for this problem.

4. PICO:

P: 45-year old male teacher with a burnout

I: CBT

C: other forms of psychotherapy

O: reduction in complaints, improvement of his functioning.

5. Search terms and results:

“Behaviour therapy” [mesh] AND "Burnout, professional"[Mesh]

72 articles, of which 13 reviews and one meta-analysis

6. Research and quality

In the meta-analysis, 48 studies were selected according to the effect of interventions in the case of ‘occupational stress’. The analysis was performed in a systematic and controlled manner. Many different outcome variables were combined, not just the functioning. It concerned only preventive interventions. It did not concern employees looking for help.

7. Results

Cognitive behaviour treatments produced the largest effect-size (0.68 95% CI from 0.54 to 0.82), followed by relaxation interventions (0.51, 95% CI from 0.33 to 0.69) and then multi-modal interventions (0.35, 95%CI from 0.22 to 0.48). All were significant. Organisation-oriented interventions had an effect-size of 0.08 (95% CI from -0.03 to 0.19), which was not significant.

8. Reference

van der Klink JJ, Blonk RW, Schene AH, van Dijk FJ. The benefits of interventions for work-related stress. *Am J Public Health*. 2001 Feb;91(2):270-6.

9. Signed

June 2003, Jos Verbeek, [j.h.verbeek@amc.uva.nl](mailto:j.h.verbeek@amc.uva.nl)

CAT IX

1. Question: Is it useful to take melatonin to prevent jetlag?

2. Answer: yes, especially when flying across several time zones in an easterly direction. Between 0,5 mg and 5 mg of melatonin is effective.

3. Background: During surgery hours of the occupational physician, a businessman who often travels to Asia for his work asks whether something can be done about the jetlag he suffers after every flight. The occupational physician has read something about melatonin and decides to look this up.

4. PICO:

P: flying in an easterly direction

I: melatonin

C: -

O: no jetlag

5. Search terms and results:

Cochrane Library: Melatonin AND jet lag

One systematic review

6. Research and quality:

Systematic review on the effectiveness of oral melatonin (in various dosages) to prevent jetlag after flights across different time zones. Nine trials have been included; all compared melatonin with a placebo, and one trial even compared it with Zolpidem (hypnotic)

7. Results

The nine trials indicate that melatonin, when taken around the normal bedtime of the destination, decreased jetlag for flights across several time zones. Daily use of melatonin between 0,5 and 5 mg have the same effect, with the exception that people fall asleep more quickly and sleep better with a dosage of 5 mg than a dosage of 0,5 mg. It is no use taking more than 5 mg. Slow-release of 2 mg tablets has no added value. NNT is 2. The benefit increases the more time zones are flown across, and if flying in an easterly direction.

The exact time at which the melatonin is taken is of importance. If taken too early, this can cause drowsiness and an extension of the time required to get accustomed to local time. Side effects do not occur often. Epilepsy and people who are taking warfarine give a counter indication.

8. Reference:

Herxheimer A, Petrie KJ Melatonin for the prevention and treatment of jet lag Herxheimer A, Petrie KJ. Melatonin for the prevention and treatment of jet lag (Cochrane Review). In: *The Cochrane Library*, Issue 2, 2004. Chichester, UK: John Wiley & Sons, Ltd.

1. Signed:
   F.G.Schaafsma, Email: [f.g.schaafsma@amc.uva.nl](mailto:f.g.schaafsma@amc.uva.nl), July 2003

CAT X

1. Question: Is St. John’s Wort more effective than placebo for an employee suffering from a mild depression?

2. Answer: St. John’s Wort is more effective than placebo and just as effective as standard anti-depressants for treating a mild depression.

3. Background: A 45-year old female teacher in secondary education is off work with complaints of depression, diagnosed by the occupational physician as mild depression. On the recommendation of a colleague, she has started drinking tea made from St. John’s Wort, which, according to the package insert, is an energy booster. The occupational physician doubts whether this is adequate therapy. Must the patient be advised to take regular anti-depressants?

4. PICO:

P: 45-year old female teacher with a depression disorder

I: St. John’s Wort

C: placebo

O: reduction of symptoms and/or improvement of functioning

5. Search terms and results:

Hypericum [Mesh] AND Depressive disorder [Mesh]

Clinical query: systematic review

6. Research and quality

In the systematic review, 47 randomized studies were summarized that met the minimum quality criteria and that all studied the effect of St. John’s Wort.

7. Results

Combining the results of 17 trials produced an OR of 2.47 (95% CI: 1.69-3.61) of St. John’s Wort versus placebo. In 10 trials the comparison with standard anti-depressants gave an OR of 1.01 (95%CI: 0.69-1.16).

8. Reference:

Linde K, Mulrow CD. St John's Wort for depression. *Cochrane Database Syst Rev*. 2000;(2):CD000448.

9. Signed

June 2003, Jos Verbeek, [J.h.verbeek@amc.uva.nl](mailto:J.h.verbeek@amc.uva.nl)

CAT XI

1. Question: Does a return to physically demanding work after an operation for lumbal hernia nuclei pulposi, six weeks after the operation, give a higher risk of a recurrence than returning to only light work?

2. Answer: heavy work does not appear to influence the chance of a recurring hernia, but recurrence is influenced by the type of hernia fragment.

3. Background: A 45-year old carpenter, six weeks after an HNP operation in which a part of the intervertebral disk was removed at the L5-S1 level, asks the occupational physician if he will ever be able to perform the work he was doing before the operation, without there being a big risk of a recurring hernia. He is experiencing a rather tired feeling in his back, but the terrible pain in his leg has totally disappeared.

4. PICO:

P: 45-year old carpenter after an operation as a result of lumbal HNP

I: physically demanding work

C: light work

O: recurring HNP

5. Search terms and results:

"Intervertebral Disk Displacement"[Mesh] AND "Recurrence"[Mesh]

Number 2 of 378 is a cohort of patients that were followed after an HNP operation.

6. Research and quality

This study concerns 187 consecutive patients operated on by a surgeon. Using logistic regression, the influence of, amongst other things, work and other confounders on the occurrence of a recurring hernia was studied. The outcome of the research was determined without knowing the operation result. Follow-up was adequately long with a median of six years.

7. Results

The most important predictor of a recurring hernia was the type of hernia fragment that was found when operating. A recurring hernia varied from 1% via 10% and 27% tot 38%. The type of work (light, medium or hard) had no influence at all on the logistic regression, but is not reported on in concrete terms of size and number.

8. Reference

Carragee EJ, Han MY, Suen PW, Kim D. Clinical outcomes after lumbar discectomy for sciatica: the effects of fragment type and anular competence. *J Bone Joint Surg Am*. 2003 Jan;85-A(1):102-8.

9. Signed

June 2003, Jos Verbeek, [j.h.verbeek@amc.uva.nl](mailto:j.h.verbeek@amc.uva.nl)

CAT XII

1. Question:

Can a male nurse, working on the ambulances, return to work two weeks after an inguinal hernia operation?

1. Answer: Yes, but in the first instance with a limit placed on physically demanding actions. For example, lifting patients. This should be possible after about three weeks.
2. Background:

A male nurse, aged 43, working on an ambulance was operated on an inguinal hernia two weeks before. He underwent inguinal hernia repair (with a mesh) and this was successful. The surgeon gave no advice with respect to returning to work. For his work, he is expected (together with a colleague) to be able to lift a sick person for a short period. He would very much like to know if he can return to work safely.

1. PICO

P: 43-year old male nurse with an inguinal hernia, I: operation, C: -, O: return to work

1. Search terms and results:

PubMed: inguinal hernia [Mesh] AND return to work / inguinal hernia AND absence from work

Results: 137 articles: three systematic reviews, various prospective studies/ eight articles; one prospective questionnaire study

1. Research and quality:
2. Systematic review: from ten different RCTs from Mesh- and non-Mesh inguinal hernia operations, the data were gathered with respect to return to work, or return to full activity, or return to normal activity.
3. Prospective study: through interviews and medical status study, data were gathered on 93 persons who had undergone various types of inguinal hernia operations within one group of surgeons. In this study, the relation between return to work and other possible variables was investigated.
4. Prospective questionnaire study: By means of a questionnaire study among 100 patients that have undergone an elective inguinal hernia operation (with a local anaesthetic) in one hospital, the duration of sickness absence was enquired about.
5. Results
6. The range in the average result between the various studies is from 4 days (limitation of daily activities) to 44 days (return to work). The conclusion in this systematic review is that a Mesh operation produces on average a more rapid return to work than another operation. The outcomes are not significant. The risk of a recurrence of an inguinal hernia varies.
7. The expected return to work period was 10 days, the actual return to work was 12 days (median 7 days, range 2-60 days) and there was no relation to state of health before the operation. Bivariate analysis reveals a relation between return to work with age, educational level, income level, profession, symptoms of depression, and the prior expectations with respect to return to work. These variables account for 61% of the range in return to work.
8. The average length of sick leave (including the day of the operation) was 8 days (range:1-16). For light or reasonably heavy work 6 days (range 3-12) and for heavy physical work 25 days (range 21-37). Pain was the most important variable for an extension of the recovery period.
9. Reference

- EU Hernia Trialists Collaboration. Mesh compared with non-mesh methods of open groin hernia repair: systematic review of randomized controlled trials. *British Journal of Surgery* 2000,87,854-859
- Jones KR, Burney RE, Peterson M, Christy B. Return to work after inguinal hernia repair. *Surgery*. 2001 Feb;129(2):128-35.
- Callesen T, Klarskov B, Bech K, Kehlet H. Short convalescence after inguinal herniorraphy with standardised recommendations: duration and reasons for delayed return to work. *Eur J Surg*. 1999 Mar;165(3):236-41

1. Signed

Frederieke Schaafsma, May 2003 Email: [f.g.schaafsma@amc.uva.nl](mailto:f.g.schaafsma@amc.uva.nl)
